# The use of social media platforms in adult basic life support research: a scoping review

**DOI:** 10.1016/j.resplu.2025.100953

**Published:** 2025-04-04

**Authors:** Nino Fijačko, Sebastian Schnaubelt, Giuseppe Stirparo, Elena Maria Ticozzi, Giuseppe Ristagno, Federico Semeraro, Robert Greif

**Affiliations:** a University of Maribor, Faculty of Health Sciences, Maribor, Slovenia; b Maribor University Medical Centre, Maribor, Slovenia; c Dpt. of Emergency Medicine, Medical University of Vienna, Austria; d PULS – Austrian Cardiac Arrest Awareness Association, Vienna, Austria; e Emergency Medical Service Vienna, Vienna, Austria; f SIMED—Società Italiana di Medicina e Divulgazione Scientifica, Parma, Italy; g AREU – Agenzia Regionale Emergenza Urgenza, Milano, Italy; h Department of Biomedical Sciences for Health, University of Milan, Milan, Italy; i Department of Pathophysiology and Transplantation, University of Milan, Italy; j Fondazione IRCCS Ca’ Granda Ospedale Maggiore Policlinico, Milan, Italy; k European Resuscitation Council, Niel, Belgium; l Department of Anesthesia, Intensive Care and Prehospital Emergency, Maggiore Hospital Carlo Alberto Pizzardi, Bologna, Italy; m Faculty of Medicine, University of Bern, Bern, Switzerland; n Department of Surgical Science, University of Torino, Torino, Italy

**Keywords:** Social media, Platforms, Adult basic life support, Application

## Abstract

**Background:**

Social media (SoMe) is expanding globally, with increasing adoption in research, including resuscitation science. Its widespread reach and growing influence make it a valuable tool for research and knowledge dissemination. We aimed to assess the utilization of SoMe, highlight its applications, and identify future research areas, specifically in data collection and analysis, education and training, and professional networking and collaboration.

**Methods:**

Embase, Scopus, and PubMed were searched through October 30th, 2024. Titles and abstracts were screened, and duplicates removed. The PCC (Population, Concept, and Context) framework defined the population as SoMe users, the concept as adult BLS-related content, and the context as SoMe platforms used for data analysis, data collection, teaching, campaigns, communication, and sharing, excluding traditional media.

**Results:**

The search yielded 5,427 articles, with 201 undergoing full-text review and 42 included. Most studies were from high-income countries (19/42; 45%) and had a cross-sectional design (16/42; 36%). SoMe was primarily used for data analysis (17/42; 41%) and data collection (16/42; 36%). YouTube and X were the frequently applied SoMe platforms (12 studies each; 29%), while Instagram and WhatsApp supported diverse applications. In contrast, Snapchat and TikTok were used less frequently and for narrower purposes.

**Conclusions:**

Existing studies focus on data collection and analysis, mainly via YouTube and X, but inconsistencies in design and geography call for standardized reporting to enhance comparability and impact. Future studies could standardize reporting on SoMe applications in adult BLS using established frameworks to ensure comparability and effectiveness.

## Introduction

As social media (SoMe) continues to evolve and gains widespread global use, its growing popularity has also been embraced by researchers of various fields including resuscitation science. As of January 2024, out of the global population of 8.8 billion, 5.6 billion had access to the internet, and 5 billion were SoMe users, reflecting a 5.6% increase compared to January 2023.^1^ Despite this widespread adoption, effectively integrating SoMe requires overcoming challenges, barriers, and limitations, including content moderation, data privacy concerns, misinformation, ethics, and the risk of information overload.^2^, ^3^, ^4^

SoMe platforms have been leveraged for public campaigns to raise awareness about cardiopulmonary resuscitation (CPR) for out of hospital cardiac arrest (OHCA), including the recent “Get Trained, Save Lives”^5^ and “European Restart a Heart Day”^6^ by the European Resuscitation Council (ERC), or the well-established “World Restart a Heart” day or week by the International Liaison Committee on Resuscitation (ILCOR).^7^ While such campaigns can reach large audiences and facilitate instant learning, evaluating their true impact in changing people’s attitude and behaviors remains challenging.^8^ At the same time, SoMe platforms hold great potential as research tools, offering opportunities to study networks, disseminate health information, and support data analysis of ongoing research related to resuscitation sciences.^9^ SoMe content was first acknowledged in the resuscitation guidelines for education nearly a decade ago^10^. In subsequent years, the guidelines offered more detailed insights into the use of specific platforms.^8^ Studies have highlighted the effectiveness of SoMe platforms in providing educational content, supporting resuscitation learning, maintaining knowledge, and identifying barriers to CPR.^8^ Our aim was to evaluate the use of SoMe in adult basic life support (BLS) research and present its potential applications, challenges, and areas for future research, specifically in data collection and analysis, education and training, and professional networking and collaboration.

## Methods

This study employed a scoping review methodology. This scoping review did not have a registered protocol; however, the review process was guided by the Preferred Reporting Items for Systematic Reviews and Meta-Analyses extension for Scoping Reviews checklist (Supplementary 1).^11^ The research question was structured using the **P**opulation of interest, **C**oncept of interest, and **C**ontext of interest (PCC) framework^12^:

**P**opulation: We defined our population as SoMe platform users, including individuals who actively engaged with SoMe platforms in various ways within the studies, as well as studies where SoMe platforms were utilized as a source of data. We categorized individuals as follows: Laypersons were categorized as individuals without formal healthcare training (e.g., high school students). Healthcare professionals were defined as individuals with a healthcare degree. Pre-licensure students referred to those currently enrolled in healthcare degree programs. We did not apply any exclusion criteria related to the population.

**C**oncept: We defined concept as the content related to adult BLS.^13^ We excluded content related to neonatal, pediatric, and advanced life support.

**C**ontext: We define the context as SoMe, which refers to: “Web-based services that allow individuals, communities, and organisations to collaborate, connect, interact, and build community by enabling them to create, co-create, modifies [sic], share, and engage with user-generated content that is easily accessible”.^14^, ^15^, ^16^ SoMe applications were categorized as follows: *Data analysis* involves analyzing SoMe content to derive insights, such as using platform data to assess public sentiment regarding OHCA. *Data collection* refers to gathering information from SoMe, for example, using SoMe platforms to distribute surveys or questionnaires. *Teaching* utilizes SoMe for educational purposes, such as sharing adult BLS content via SoMe platforms. *Campaigns* focus on promoting initiatives or raising awareness, like educating the public about CPR. *Communication* involves using SoMe platforms for direct interactions with researchers, while *sharing* pertains to exchanging messages, information, or user-generated content. We excluded traditional media, including newspapers, television and radio.

Additionally, we selected studies based on their design and time of publication.

Study design: Randomized controlled trials, nonrandomized (controlled) studies, controlled before-and-after studies, cohort studies, case series and research letters were eligible for inclusion.^17^ Publications in English, Italian, Slovenian and Spanish were included. Reviews, conference abstracts, registered trials, editorials, letters to the editor, and notes were excluded manually or using search hedges and/or removed during screening.

Timeframe: 1960 – January 1st, 2025.

Part of the authors (NF, GS, ELM) developed a search strategy based on methodologies from similar reviews^18^, ^19^ on SoMe in healthcare research and a standard search syntax for health sciences databases.^20^ The databases searched included Embase, Scopus, and PubMed, with detailed strategies for each outlined in Supplementary 2. Additionally, manual reference searching (NF) was performed to identify further relevant studies.

Search results were exported into Rayyan Intelligent Systematic Review software (Qatar Computing Research Institute, Doha, Qatar), where duplicates were removed. Titles and abstracts were screened independently by three pairs of reviewers (NF & TG; SS & FS; GS & ELM) who found consensus. In case of conflicts a third reviewer (FS or NF) resolved it. Full-text assessment was conducted by NF & TG, and SS resolved any conflicts.

The final data extraction captured key information, including the surname of the first author, publication year, the first author's country of origin, the income classification of the first author's country, and PCC. The income classification of the first author's country was take from the World Bank.^21^ Data from SoMe studies were organized using Microsoft Excel 365 (Microsoft Corporation, USA), and visualized with a PRISMA flow diagram^22^ and the Sankeymatic tool (Sankeymatic, USA).

## Results

**Fig. 1:**
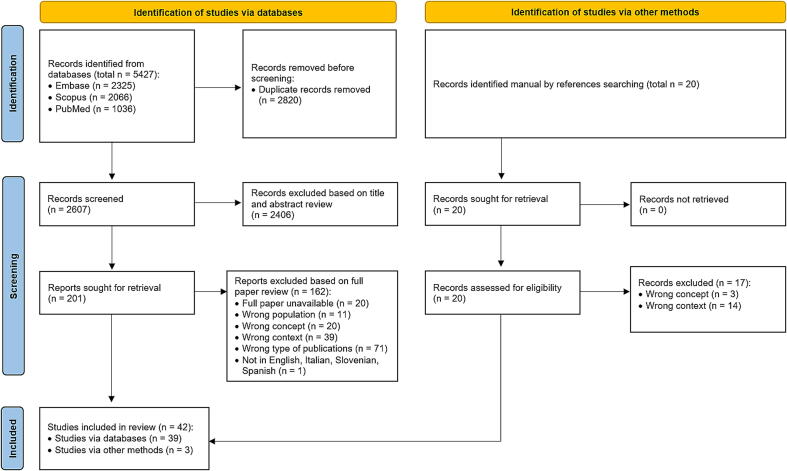
Flow diagram of selection process.

Our search was performed on October 30th, 2024. A total of 5,427 records were identified (Embase (*n* = 2,325), Scopus (*n* = 2,066), and PubMed (*n* = 1,036); 2,820 duplicates were removed, leaving 2,607 records for title and abstract screening, which excluded an additional 2,406 records. As a result, 201 articles underwent full-text assessment, which excluded other 160 with the following reasons: no full text available (*n* = 20), wrong population (*n* = 11), wrong concept (*n* = 20), wrong context (*n* = 39), incorrect publication type (*n* = 71), and non-English or Italian, Slovenian and Spanish full text (*n* = 1). We included three more articles through a manual reference screening.^23^, ^24^, ^25^ Finally, 42 articles were included in the scoping review (Fig. 1).^23^, ^24^, ^25^, ^26^, ^27^, ^28^, ^29^, ^30^, ^31^, ^32^, ^33^, ^34^, ^35^, ^36^, ^37^, ^38^, ^39^, ^40^, ^41^, ^42^, ^43^, ^44^, ^45^, ^46^, ^47^, ^48^, ^49^, ^50^, ^51^, ^52^, ^53^, ^54^, ^55^, ^56^, ^57^, ^58^, ^59^, ^60^, ^61^, ^62^, ^63^, ^64^

The included studies spanned from 2011 to 2025, with ten studies published in 2024^23^, ^24^, ^57^, ^58^, ^59^, ^60^, ^61^, ^62^, ^63^, ^64^. All included studies were written in English (*n* = 41)^23^, ^24^, ^25^, ^26^, ^27^, ^28^, ^29^, ^30^, ^31^, ^32^, ^33^, ^34^, ^35^, ^37^, ^38^, ^39^, ^40^, ^41^, ^42^, ^43^, ^44^, ^45^, ^46^, ^47^, ^48^, ^49^, ^50^, ^51^, ^52^, ^53^, ^54^, ^55^, ^56^, ^57^, ^58^, ^59^, ^60^, ^61^, ^62^, ^63^, ^64^, except for one in Spanish.^36^

Based on the country income level of the first authors of origin, 19 articles came from high-income countries (45%)^23^, ^25^, ^26^, ^36^, ^44^, ^48^, ^50^, ^51^, ^53^, ^57^, ^64^, ^28^, ^29^, ^30^, ^31^, ^32^, ^39^, ^40^, ^41^, 16 from upper-middle countries (38%)^24^, ^27^, ^38^, ^42^, ^43^, ^47^, ^54^, ^56^, ^33^, ^34^, ^35^, ^58^, ^59^, ^60^, ^61^, ^62^, six from lower-middle income countries (14%)^37^, ^45^, ^46^, ^52^, ^55^, ^63^, and one from a low-income country (2%)^49^. Sixteen studies had a cross-sectional design (36%)^24^, ^25^, ^36^, ^37^, ^39^, ^44^, ^45^, ^47^, ^49^, ^51^, ^52^, ^54^, ^55^, ^57^, ^60^, ^64^, 10 studies used comparative analysis (24%)^33^, ^41^, ^42^, ^48^, ^50^, ^56^, ^58^, ^26^, ^27^, ^28^, nine studies were quasi-experimental (21%)^34^, ^35^, ^38^, ^43^, ^46^, ^59^, ^61^, ^62^, ^63^, and six studies had a retrospective design (17%).^40^, ^53^, ^29^, ^30^, ^31^, ^32^ Sixteen studies used SoMe platforms for data analysis (38%)^23^, ^41^, ^42^, ^48^, ^50^, ^53^, ^54^, ^56^, ^58^, ^26^, ^27^, ^28^, ^30^, ^31^, ^32^, ^33^, 13 focused on laypersons (29%)^24^, ^29^, ^34^, ^36^, ^37^, ^39^, ^40^, ^44^, ^47^, ^57^, ^60^, ^63^, ^64^, nine involved pre-licensure students (22%)^25^, ^38^, ^43^, ^46^, ^49^, ^52^, ^59^, ^61^, ^62^, three included healthcare professionals (7%),^35^, ^51^, ^55^ and one involved both healthcare professionals and laypersons (2%) (Table 1).^45^

**Table 1:** Characteristics of included SoMe studies, reported in chronological order.

**SoMe studies**	**Countries income**^21^	**Type of study**	**Population**
1. Murugiah, et al., 2011,^26^ (USA)	High income	Comparative analysis	SoMe data
2. Tourinho, et al., 2012,^27^ (Brazil)	Upper middle income	Comparative analysis	SoMe data
3. Boslye, et al., 2013,^28^ (USA)	High income	Comparative analysis	SoMe data
4. Plunien, et al., 2017,^29^ (Germany)	High income	Retrospective study	Laypersons
5. Leary, et al., 2018,^30^ (USA)	High income	Retrospective study	SoMe data
6. McGovern, et al., 2018,^31^ (USA)	High income	Retrospective study	SoMe data
7. Jalali, et al., 2019,^32^ (USA)	High income	Retrospective study	SoMe data
8. Katipoglu, et al., 2019,^33^ (Turkey)	Upper middle income	Comparative analysis	SoMe data
9. Xi, and Tan, 2019,^34^ (China)	Upper middle income	Quasi-experimental study	Laypersons
10. Zia Ziabari, et al., 2019,^35^ (Iran)	Upper middle income	Quasi-experimental study	Healthcare professionals
11. Alvarez-Cebreiro, et al., 2020,^36^ (Spain)	High income	Cross-sectional study	Laypersons
12. Anto-Ocrah, et al., 2020,^37^ (Ghana)	Lower middle income	Cross-sectional study	Laypersons
13. Ghorbani, et al., 2020,^38^ (Iran)	Upper middle income	Quasi-experimental study	Pre-licensure students
14. Grunau, et al., 2020,^39^ (Canada)	High income	Cross-sectional study	Laypersons
15. Böttiger, et al., 2020,^40^ (Germany)	High income	Retrospective study	Laypersons
16. Lynes, and Toft, 2020,^41^ (USA)	High income	Comparative analysis	SoMe data
17. Yilmaz Ferhaoglu and Kudsioglu, et al., 2020,^42^ (Turkey)	Upper middle income	Comparative analysis	SoMe data
18. Farsi, et al., 2021,^43^ (Iran)	Upper middle income	Quasi-experimental study	Pre-licensure students
19. Chilappa, and Waxman, 2021,^44^ (USA)	High income	Cross-sectional study	Laypersons
20. Albazee, et al., 2022,^45^ (Jordan, Egypt)	Lower middle income	Cross-sectional study	Healthcare professionals, laypersons
21. Chaudhuri, et al., 2022,^46^ (India)	Lower middle income	Quasi-experimental study	Pre-licensure students
22. Akhagbaker and Aziz, et al., 2022,^47^ (Iraq)	Upper middle income	Cross-sectional study	Laypersons
23. Ferrell, et al., 2022,^48^ (USA)	High income	Comparative analysis	SoMe data
24. Ssewante, et al., 2022,^49^ (Uganda)	Low income	Cross-sectional study	Pre-licensure students
25. Vilela, et al., 2022,^50^ (Spain)	High income	Comparative analysis	SoMe data
26. Alomi, et al., 2023,^51^ (Saudi Rabia)	High income	Cross-sectional study	Healthcare professionals
27. Alkarrash, et al., 2023,^52^ (Egypt)	Lower middle income	Cross-sectional study	Pre-licensure students
28. Fijačko, et al., 2023,^53^ (Slovenia)	High income	Retrospective study	SoMe data
29. Gezer, et al., 2023^54^m (Turkey)	Upper middle income	Cross-sectional study	SoMe data
30. Onabanjo, et al., 2023,^55^ (Nigeria)	Lower middle income	Cross-sectional study	Healthcare professionals
31. Topcu, et al., 2023,^56^ (Turkey)	Upper middle income	Comparative analysis	SoMe data
32. Rosa-Castillo, et al., 2023,^25^ (Spain)	High income	Cross-sectional study	Pre-licensure students
33. Sayed, et al., 2024,^57^ (Saudi Arabia)	High income	Cross-sectional study	Laypersons
34. Aksoy, 2024,^58^ (Turkey)	Upper middle income	Comparative analysis	SoMe data
35. Falahan, et al., 2024,^59^ (Iran)	Upper middle income	Quasi-experimental study	Pre-licensure students
36. Gao, et al., 2024,^60^ (China)	Upper middle income	Cross-sectional study	Laypersons
37. Katapadi, et al., 2024,^64^ (USA)	High income	Cross-sectional study	Laypersons
38. Miri, et al., 2024,^61^ (Iran)	Upper middle income	Quasi-experimental study	Pre-licensure students
39. Ranjbar, et al., 2024,^62^ (Iran)	Upper middle income	Quasi-experimental study	Pre-licensure students
40. Ravindra, et al., 2024,^63^ (India)	Lower middle income	Quasi-experimental study	Laypersons
41. Tam, and Kwok, 2024,^24^ (China)	Upper middle income	Cross-sectional study	Laypersons
42. Choi, et al., 2025,^23^ (South Korea)	High income	Retrospective study	SoMe data

Table 2 presents six applications of SoMe platforms usage: 1) Data analyses, 2) Data collection, 3) Teaching, 4) Campaigns, 5) Communication and 6) Sharing content. Data analyses were the most common application of SoMe platforms usage (17 studies, 41%)^23^, ^41^, ^42^, ^46^, ^48^, ^50^, ^53^, ^54^, ^56^, ^58^, ^26^, ^27^, ^28^, ^30^, ^31^, ^32^, ^33^, followed data collection (16 studies, 36%)^24^, ^34^, ^37^, ^38^, ^44^, ^45^, ^47^, ^49^, ^51^, ^52^, ^55^, ^57^, ^64^, ^60^, ^61^, ^62^, teaching (four studies, 10%)^25^, ^35^, ^36^, ^38^, campaigns (three studies, 7%)^29^, ^40^, ^63^, communication^43^ and sharing,^59^ with one study each (4%). Many of SoMe platforms included in the studies originated from the USA (Instagram, Facebook, WhatsApp – Meta Platforms, Inc.; Snapchat – Snap Inc.; YouTube – Google LLC; X [formerly Twitter] – X Corp.). Others were from China (TikTok – ByteDance Ltd., WeChat – Tencent Holdings Ltd.) and the British Virgin Islands (Telegram – Telegram Messenger Inc.). YouTube^23^, ^26^, ^27^, ^33^, ^41^, ^42^, ^46^, ^47^, ^50^, ^54^, ^56^, ^58^ and X^36^, ^39^, ^45^, ^48^, ^53^, ^55^, ^57^, ^64^, ^28^, ^29^, ^30^, ^31^ were the most frequently used SoMe platform (each 12 studies; 29%). Less common were Snapchat (three studies; 7%),^44^, ^47^, ^64^ and TikTok (one study; 2%).^64^ The number of SoMe platforms used per study varies from one to six, with one being the most common (Table 2).

**Table 2:** SoMe platforms and their applications.

**SoMe studies**		**SoMe platforms**									
	**Application of** **SoMe platforms usage**	**Instagram**	**Telegram**	**Facebook**	**X**	**YouTube**	**WhatsApp**	**WeChat**	**Snapchat**	**TikTok**	**Total**
1. Murugiah, et al., 2011,^26^ (USA)	Data analyses					X					1
2. Tourinho, et al., 2012,^27^ (Brazil)	Data analyses					X					1
3. Boslye, et al., 2013,^28^ (USA)	Data analyses				X						1
4. Plunien, et al., 2017,^29^ (Germany)	Campaign			X							1
5. Leary, et al., 2018,^30^ (USA)	Data analyses				X						1
6. McGovern, et al., 2018,^31^ (USA)	Data analyses				X						1
7. Jalali, et al., 2019,^32^ (USA)	Data analyses				X						1
8. Katipoglu, et al., 2019,^33^ (Turkey)	Data analyses					X					1
9. Xi, and Tan, 2019,^34^ (China)	Data collection							X			1
10. Zia Ziabari, et al., 2019,^35^ (Iran)	Teaching		X								1
11. Alvarez-Cebreiro, et al., 2020,^36^ (Spain)	Teaching			X	X		X				3
12. Anto-Ocrah, et al., 2020,^37^ (Ghana)	Data collection			X				X			2
13. Ghorbani, et al., 2020,^38^ (Iran)	Teaching							X			1
14. Grunau, et al., 2020,^39^ (Canada)	Data collection	X		X	X						3
15. Böttiger, et al., 2020,^40^ (Germany)	Campaign	X		X							2
16. Lynes, and Toft, 2020,^41^ (USA)	Data analyses					X					1
17. Yilmaz Ferhaoglu and Kudsioglu, et al., 2020,^42^ (Turkey)	Data analyses					X					1
18. Farsi, et al., 2021,^43^ (Iran)	Communication		X								1
19. Chilappa, and Waxman, 2021,^44^ (USA)	Data collection								X		1
20. Albazee, et al., 2022,^45^ (Jordan, Egypt)	Data collection			X	X		X				3
21. Chaudhuri, et al., 2022,^46^ (India)	Data analyses					X	X				2
22. Akhagbaker and Aziz, et al., 2022,^47^ (Iraq)	Data collection	X	X	X		X	X		X		6
23. Ferrell, et al., 2022,^48^ (USA)	Data analyses				X						1
24. Ssewante, et al., 2022,^49^ (Uganda)	Data collection						X				1
25. Vilela, et al., 2022,^50^ (Spain)	Data analyses					X					1
26. Alomi, et al., 2023,^51^ (Saudi Rabia)	Data collection		X				X				2
27. Alkarrash, et al., 2023,^52^ (Egypt)	Data collection		X	X			X				3
28. Fijačko, et al., 2023,^53^ (Slovenia)	Data analyses				X						1
29. Gezer, et al., 2023,^54^ (Turkey)	Data analyses					X					1
30. Onabanjo, et al., 2023,^55^ (Nigeria)	Data collection		X		X		X				3
31. Topcu, et al., 2023,^56^ (Turkey)	Data analyses					X					1
32. Rosa-Castillo, et al., 2023,^25^ (Spain)	Teaching	X									1
33. Sayed, et al., 2024,^57^ (Saudi Arabia)	Data collection			X	X		X				3
34. Aksoy, 2024,^58^ (Turkey)	Data analyses					X					1
35. Falahan, et al., 2024,^59^ (Iran)	Sharing the content						X				1
36. Gao, et al., 2024,^60^ (China)	Data collection							X			1
37. Katapadi, et al., 2024,^64^ (USA)	Data collection	X		X	X				X	X	5
38. Miri, et al., 2024,^61^ (Iran)	Data collection		X								1
39. Ranjbar, et al., 2024,^62^ (Iran)	Data collection		X								1
40. Ravindra, et al., 2024,^63^ (India)	Campaign	X									1
41. Tam, and Kwok, 2024,^24^ (China)	Data collection	X		X			X				3
42. Choi, et al., 2025,^23^ (South Korea)	Data analyses	X				X					2
**Total:**		8	8	11	12	12	11	4	3	1	

**Fig. 2:**
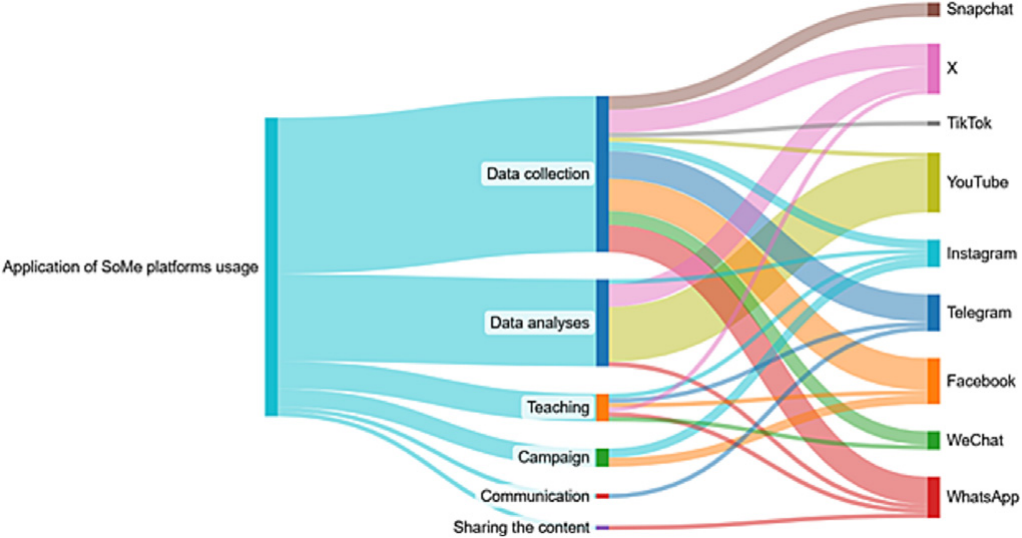
Distribution of SoMe platforms across different types of usage.

Instagram and WhatsApp stood out as the most versatile platforms, supporting a wide application of SoMe usages, whereas platforms like Snapchat or TikTok were used more narrowly and less frequently. Platforms such as Facebook, WhatsApp, X, and Telegram were predominantly applied for data collection, while YouTube and X were frequently applied for data analyses (Fig. 2).

**Fig. 3:**
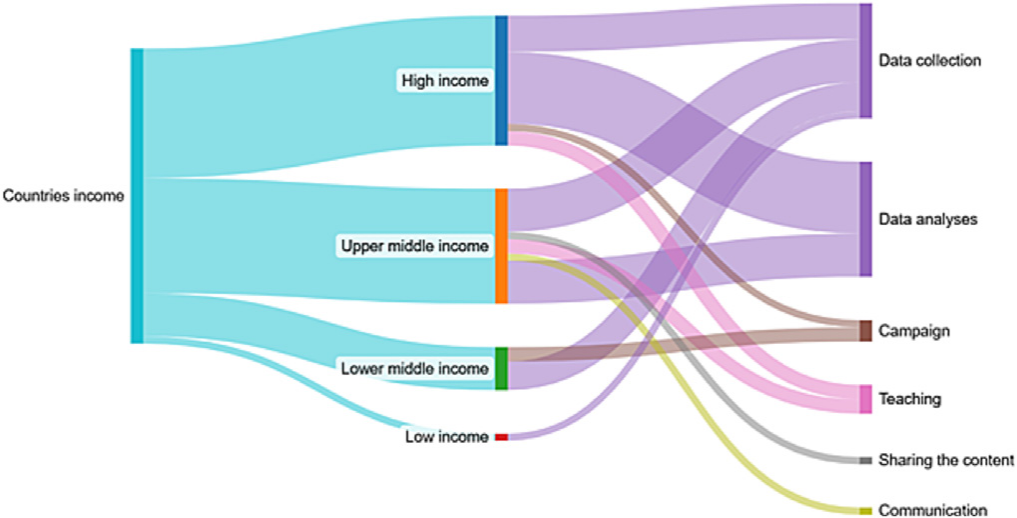
Relationship between countries' income and application of SoMe platforms usage.

High- and upper-middle-income countries applied SoMe platforms for data collection and analysis, while lower-middle-income countries focused more on data collection and campaigns. Additionally, high- and upper-middle-income countries applied to SoMe platforms for teaching, whereas upper-middle-income countries also applied them for content sharing and communication (Fig. 3).

## Discussion

Our review underscored the broad and varied application of SoMe in adult BLS research, spanning over diverse populations, concepts, and contexts of interest.

We revealed that YouTube,^23^, ^26^, ^27^, ^33^, ^41^, ^42^, ^46^, ^47^, ^50^, ^54^, ^56^, ^58^ and X^23^, ^26^, ^27^, ^33^, ^41^, ^42^, ^46^, ^47^, ^50^, ^54^, ^56^, ^58^ were the most applied SoMe platforms for adult BLS-related research activities, while platforms such as Snapchat^44^, ^47^, ^64^ and TikTok^64^ were less frequently employed. This pattern likely reflects the historical dominance of these platforms concerning information dissemination and their user demographics.^65^, ^66^ Although, emerging platforms like LinkedIn (LinkedIn, USA), Bluesky (Bluesky Social PBC, USA), Mastodon (Mastodon GmbH, Germany), and Bilibili (Bilibili Inc., China) have been gaining prominence, they were not identified in our review. For instance, Bluesky and Mastodon are being recognised as a viable alternative to X for professional engagement,^67^, ^68^ while Bilibili is emerging as a counterpart to YouTube and TikTok, particularly among younger users and in East Asian regions.^69^, ^70^, ^71^ The diversification of platform usage underscores the evolving landscape of SoMe and the need for adaptable strategies to maximize their potential in adult BLS research. For instance, LinkedIn supports professional networking, research sharing, and adult BLS webinars, while Bluesky fosters decentralized discussions, feedback, and advocacy. Mastodon facilitates open-access research sharing and specialized adult BLS discussions, and Bilibili is ideal for engaging educational videos and adult BLS demonstrations. Utilizing these platforms enhances adult BLS research, training, and outreach.

Our results indicate that SoMe has primarily been employed for data analysis,^23^, ^41^, ^42^, ^46^, ^48^, ^50^, ^53^, ^54^, ^56^, ^58^, ^26^, ^27^, ^28^, ^30^, ^31^, ^32^, ^33^ particularly analysing user-generated content on X^53^, ^29^, ^30^, ^31^ or educational materials on YouTube.^26^, ^27^, ^41^, ^42^, ^50^, ^56^, ^58^, ^64^ Notably, X has frequently been used to study laypersons' feelings about witnessing sudden cardiac arrest, including two recent instances involving sports fans in the Europe and in the USA.^48^, ^53^, ^72^, ^73^, ^74^, ^75^ SoMe platforms that focus on video content (e.g., YouTube, Bilibili, TikTok) have significant potential for teaching health related content to general populations.^70^, ^71^ However, ensuring expert validation of adult BLS content is crucial, as this review highlights that many platforms, including YouTube, feature content that does not fully adhere to BLS guidelines.^26^, ^27^, ^41^, ^42^, ^50^, ^56^, ^58^, ^64^ The varying use of platforms for educational purposes may be influenced by factors such as content regulation, accessibility, and user engagement. On the other hand, short video content about adult BLS has been shown to enhance responsiveness and performance among laypersons.^76^, ^77^

Our review demonstrated that SoMe platforms were less commonly used for teaching adult BLS content compared to other applications. However, Instagram, Telegram, X, WeChat, WhatsApp, and Facebook have been used for teaching adult BLS, but with significant heterogeneity in their application,^25^, ^35^, ^36^, ^38^ due to variability in content quality, study design, and geographic representation. SoMe holds significant potential as a teaching tool, particularly for laypersons such as family members of cardiovascular patients.^34^, ^78^ This aligns with the recently proposed updates to the chain of survival, where family members of out-of-hospital cardiac arrest victims are being recognized as an integral link within the chain of survival.^79^, ^80^

We presented that high- and upper-middle-income countries primarily used SoMe platforms for data collection and analysis. Lower-middle-income countries also focused on data collection but with a stronger emphasis on campaigns. In low-income countries, SoMe use for data collection was minimal, likely due to limited internet access, digital infrastructure, and technological resources.^81^, ^82^ These limitations create inequalities in access to information and can introduce significant biases in data analysis.^48^ In terms of data collection, when distributing questionnaires through SoMe channels, researchers could consider the phenomenon of homophily, defined as “the tendency for individuals to seek out or be drawn to others who share similar characteristics”. According to this principle, individuals who follow scientific sources on SoMe, and are therefore reached by data collection questionnaires, are likely to be more invested in these topics than the general population.^58^, ^83^ As a result, responses to electronic surveys distributed through SoMe may present some limitations, as they are more likely to come from individuals with prior knowledge, specific attitudes, or increased interest in the subject. Indeed, people with a scientific background tend to follow and interact with popular sources on SoMe that cover scientific topics in line with their interests, and on many SoMe the algorithm will continue to suggest this content to keep them engaged. These selection biases may lead to an over-representation of this population, potentially limiting the generalizability of the findings.

In addition, the versatility of SoMe platforms extends beyond education and data analysis to foster global collaboration. Platforms such as LinkedIn and Mastodon, which are designed to facilitate professional networking,^67^, ^68^ may serve as emerging hubs for both interdisciplinary and multidisciplinary collaboration in adult BLS research. Leveraging these platforms could bridge gaps between researchers, educators, and policymakers, promoting broader collaboration and enhancing the dissemination and implementation of resuscitation guidelines worldwide.^84^

SoMe platforms that offer a wide range of applications, including text messaging, voice messaging, broadcast messaging, video conferencing, video games, photo and video sharing, location sharing, and payment services, are referred to as super apps,^85^ with WeChat, X and WhatsApp being a notable example.^86^, ^87^ Furthermore, new applications in SoMe platforms, such as augmented reality filters in Instagram or Snapchat, have been developed and tested for teaching adult BLS content^83^, ^88^ However, none of these innovations have yet been part of the research which could be found in hierarchy of scientific evidence.^17^ As one example, an Instagram-based educational game demonstrated better learning outcomes for nursing students compared to their counterparts who did not participate in the game.^25^ Emerging platforms and technologies including super apps and extended reality offer new and exciting opportunities for adult BLS education.

Although SoMe platforms are a useful resource, there are some limitations that need to be addressed. Importantly, their content changes over time, making it difficult to obtain a comprehensive view of their content over a long time period.^48^, ^50^ In addition, several SoMe platforms adopt models where shared content is only available to friends or subscribers (e.g. Instagram, Facebook, YouTube), creating a barrier to data accessibility for research purposes.^25^, ^50^

Another potential bias arises from the fact that different SoMe attract users according to their specific interests and are therefore unlikely to be representative of the general population, but rather of specific subgroups.^50^, ^53^, ^73^ Several studies have highlighted the lack of quality control systems for uploaded content, making it a double-edged weapon, as it can spread false or incomplete information that may be harmful, leading to a lack of trust in health professionals or the adoption of dangerous practices.^32^, ^41^, ^49^, ^53^ Meta Platforms, Inc. has announced plans to implement a “Community Notes” system across its platforms, including Facebook, and Instagram. This initiative is inspired by a similar feature on X and aims to enhance content moderation by leveraging user contributions. The system allows selected users to add contextual notes to posts they consider misleading or lacking context. These notes become visible to the broader user base once they receive sufficient positive ratings from contributors with diverse perspectives. This approach is intended to promote free expression while reducing reliance on traditional fact-checking methods.^89^, ^90^ While this system fosters community-driven moderation, it also raises ethical concerns regarding the potential for bias, misinformation amplification, and the disproportionate influence of certain user groups. Ensuring that contributions represent a balanced range of perspectives remains a challenge. Additionally, the handling of user-generated content introduces data confidentiality risks. SoMe platforms must implement robust safeguards to prevent unauthorized data access, ensure contributor anonymity where necessary, and comply with privacy regulations. Striking a balance between transparency and protecting user data will be crucial for maintaining trust in the system.

## Limitations

Our study has three key limitations. First, based on our inclusion criteria, we did not include databases such as PsycInfo, which focuses more on psychology, due to limited access to these resources. Second, we focused exclusively on adult BLS, leaving neonatal, pediatric, and advanced life support for future work. Third, we excluded studies published in Chinese, which may explain the limited number of studies utilizing WeChat. To address the exclusion of Chinese-language studies, future research could include linguistically diverse researchers. Lastly, this review did not include ResearchGate, as it primarily serves academic networking rather than broad public engagement like other SoMe platforms analyzed. Future research could assess its role in data sharing and collaboration.

## Conclusion

To our knowledge, this is the first review examining the use of SoMe in adult BLS research. While existing studies primarily focus on data collection and analysis, particularly through platforms like YouTube and X, the lack of consistency in study design, methodology, and geographic representation limits the generalizability of findings. The heterogeneity in how different SoMe platforms are utilized for education, training, and research further underscores the need for a standardized approach. The need for expert validation of BLS-related content is evident, as much of the existing material does not align with current guidelines. Future studies could adopt established frameworks to systematically report SoMe applications in adult BLS, ensuring greater comparability, reliability, and effectiveness in leveraging these platforms for resuscitation education and research.

## Conflicts of interest

NF is a member of the European Resuscitation Council (ERC) Basic Life Support (BLS) Science and Education Committee. SS is member of the ILCOR Education, Implementation and Teams (EIT) Task Force EIT and ERC Advanced Life Support SEC ALS. GR is ERC Director of Congresses, ILCOR BLS Task Force Emeritus member, and a vice President of the Italian Resuscitation Council Foundation. FS is the Chair of the ERC, ILCOR BLS Task Force Emeritus member, and a member of the Italian Resuscitation Council Foundation. RG is ERC Director of Guidelines and ILCOR, and ILCOR Task Force chair for Education Implementation and Team; and member of the editorial board of Resuscitation Plus. Other authors (GS, EMT) declare that they have no conflict of interest.

## CRediT authorship contribution statement

**Nino Fijačko:** Writing – review & editing, Writing – original draft, Visualization, Supervision, Methodology, Investigation, Formal analysis, Data curation, Conceptualization. **Sebastian Schnaubelt:** Writing – review & editing, Methodology, Investigation, Formal analysis, Data curation. **Giuseppe Stirparo:** Writing – review & editing, Methodology, Investigation, Formal analysis, Data curation. **Elena Maria Ticozzi:** Writing – review & editing, Methodology, Investigation, Formal analysis, Data curation. **Giuseppe Ristagno:** Writing – review & editing, Resources, Funding acquisition, Data curation. **Federico Semeraro:** Writing – review & editing, Methodology, Investigation, Formal analysis, Data curation, Conceptualization. **Robert Greif:** Writing – review & editing, Methodology, Investigation, Formal analysis, Data curation.

## Funding

This study was funded in part by Italian Ministry of Health − Current research IRCCS and by linea 2 University of Milan to GR.

## Declaration of competing interest

The authors declare that they have no known competing financial interests or personal relationships that could have appeared to influence the work reported in this paper.
